# Amelioration of lipid production by *Cunninghamella echinulata*: statistical optimization and GC-MS characterization

**DOI:** 10.1186/s12934-026-03134-3

**Published:** 2026-11-03

**Authors:** Hany A. A. Abdou, Mohamed A. Abdel-Sater, Abdel-Rehim A. A. El-Shanawany, Abdallah M. A. Hassane

**Affiliations:** 1 Department of Botany and Microbiology, Faculty of Science, Assiut Branch, Al-Azhar University, Assiut, 71524 Egypt; 2 Department of Botany and Microbiology, Faculty of Science, Assiut University, Assiut, 71515 Egypt

**Keywords:** Oleaginous fungi, *Cunninghamella echinulata*, Optimization, Agro-wastes, GC-MS, Antimicrobial

## Abstract

Lipids have garnered attention for their importance in pharmaceuticals and nutraceuticals. Famously, oleaginous filamentous fungi produce essential fatty acids. In this study, optimization using one factor at time (OFAT) and response surface methodology-Box-Behnken design (RSM-BBD) approaches, *Cunninghamella echinulata* showed the highest lipid content (61.23%) under optimized OFAT conditions (60 g/L, glucose; 2 g/L; ammonium tartrate; pH value, 7 at 30 °C, and shaking for seven days). Lipid content of 59.37% was achieved using the BBD. One-way ANOVA indicated temperature significantly affected lipid content, while two-way ANOVA revealed significant interactions between temperature and ammonium tartrate concentration, pH and incubation time, and inoculum size and glucose concentration. Various agro-wastes used as media for *C. echinulata* growth and production of lipid with peach yielding the best lipid content (49.30%). Comparative analysis of lipid extracts by GC-MS of the best yield conditions revealed nineteen different fatty acids, with oleic acid, linoleic acid, gamma-linolenic acid, and palmitic acid present in high percentages of 35.49%, 21.31%, 21.78%, and 13.44%, respectively. The extracted lipids demonstrated antibacterial potential towards *Staphylococcus aureus*, *Escherichia coli*, and *Bacillus subtilis*, as well as antifungal efficacy against *Candida albicans* and *C. glabrata*. The inhibition zones ranged from 9 to 19 mm with 50 µL of 10% extracts (100 mg/mL). This investigation revealed that optimization of *C. echinulata* represents promising candidate for high lipid yield in addition to lipid extracts may serve as potential antimicrobial agents in the pharmaceutical industry.

**Supplementary Information:**

The online version contains supplementary material available at https://doi.org/10.1186/s12934-026-03134-3.

## Introduction

Oleaginous microorganisms represent a group of species belonged to diverse genera, including fungi, yeasts, algae, and bacteria [1, 2]. These microorganisms can accumulate high lipid content (over 20% of the dry weight of their cells) within their cellular compartments [3]. Fungi are a significant group of microorganisms capable of producing single-cell oils because of their short life cycle, independence from light energy, and their capacity to use a variety of carbon sources, including agro-industrial residues, crude glycerol, and wastewater among others [4]. Various fungal species, including *Mortierella isabellina*, *M. vinacea*, *Rhizopus oryzae*, *Mucor circinelloides*, *Cunninghamella echinulata*, *Aspergillus oryzae*, and *A. terreus* have identified as lipid producers [5].

After proteins and carbohydrates, lipids are the third most abundant class of biomacromolecules found in living organisms [6] where cellular membranes, energy sources, signaling routes, hormones, vitamins, and gene expression are all biologically dependent on lipids [7]. Fatty acids, saturated (SFAs), monounsaturated fatty acids (MUFAs), and poly-unsaturated (PUFAs), are principal constituents of membrane lipids and for human nutrition in addition to many health benefits for the human body [8]. Traditionally, PUFAs are derived from plants and animals; however they can be also obtained from oleaginous microorganisms like fungi that are sustainable and independent of climatic or seasonal changes [9].

In the stationary phase, lipid droplets accumulate in fungi as nitrogen depletes and carbon levels rise. Various factors, including carbon/nitrogen (C/N) ratio, incubation temperature, pH value, incubation time, agitation, and substrate concentrations, influence this accumulation [9, 10]. The C/N ratio in culture medium represents one of the most important nutritional elements impacting the total lipids yield because it controls the switch between protein and lipid biosynthesis [11]. Also, for oleaginous organisms to accumulate lipids, the C/N ratio should be greater than 20 [12]. The citric acid cycle is down regulated as a result of lipid buildup, which begins in reaction to a stress environment (nitrogen restriction). Because cellular reproduction is inhibited, there is an increase in cell dry weight and high lipid content, and the resulting lipid bodies serve as a source of energy for already-existing cells [13, 14]. Because cell development fluctuates, the lipid composition is dependent on the N source used in the culture medium. Lipid accumulation is also affected when organic sources are utilized rather than inorganic ones [15].

Monitoring the fatty acid content of fungus, which affects the degree of unsaturation, depends on temperature. Different optimal temperatures affect the saturation degree in the conformation of triacylglycerols because they need unique enzyme activity to occur in lipid metabolism [16, 17]. Low temperatures inhibit fungal growth and lipid accumulation [18], while moderate temperatures, ranging from 25 °C to 30 °C, favor high cell cultivation across various oleaginous fungal species [19]. Lipid yields and cell proliferation were significantly influenced by the pH value fluctuation [9]. Notably, pH affects nutrients availability needed for growth of fungus and lipid buildup as well as the activity of enzymes involved in lipid biosynthesis [20].

Lipid accumulation in oleo-microbes is influenced by molasses, sugar beet, watermelon peel, rotten fruits, soluble starch, and hydrolyzable carbohydrates such glucose, sucrose, lactose, and raffinose [21, 22]. Lipid biosynthesis can be significantly impacted by the kind of substrate used for fungal growth. Precursor’s availability for biosynthesis of lipid and the involved metabolic pathways might be affected by various carbon and nitrogen sources [23–25]. The present study aimed to optimize the oleaginous soil *C. echinulata* for lipid accumulation employing OFAT, RSM-BBD, and waste conditions. The FAs profile of extracted lipids was characterized by GC-MS and their antimicrobial potential was investigated. The present work relies on the integration of several aspects in one single study, namely the optimisation of lipid biosynthesis, the evaluation of low-cost agro-industrial substrates as alternative feed stocks and the assessment of antimicrobial activity of extracted lipids. There are few studies, to our knowledge, that have combined these approaches for *C. echinulata*.

## Materials and methods

### Fungal isolate

*Cunninghamella echinulata* (Thaxt.) Thaxt. ex Blakeslee was isolated from agriculture soil (Campus of Al-Azhar University, Assiut, Egypt) on PDA medium in our previous study by Abdou et al. [26].

### Molecular identification of fungal isolate

Total DNA was extracted from pure fungal culture, cultivated in Czapeks’ broth for 5 days at 28 °C, by “Norgen Plant/Fungi DNA Isolation Kit (Sigma, Thorold, Canada)” and preserved at -20 °C [27]. According to Mohamed et al. [28], internal transcribed spacer (ITS) region of the tested strain was amplified using specific universal primers ITS-1 (5′-TCC GTA GGT GAA CCT GCG G-3′) and ITS-4 (5′-TCC TCC GCT TAT TGA TAT GC-3′), while PCR reaction was performed as described previously by Hassan et al. [27]. The obtained sequences were aligned with the BLAST search tool at (NCBI) to expose their similarity. BioEdit software program version No. 7.2.5. was utilized to inspect and analyze the sequences of ITS regions, while the search of homology was performed by comparing with strains obtained from the sequencing databases by using a BLAST search algorithm of the GenBank (http://www.ncbi.nlm.nih.gov/BLAST/).

### Screening for lipid production and cultivation conditions

To induce lipid accumulation, lipid fermentation medium was utilized with nitrogen limitation and carbon richness. One-hundred mL of sterilized Kendrick and Ratledge (K & R) broth medium (composed of g/L: (30 g) glucose, (3.3 g) ammonium tartrate, (7 g) KH_2_PO_4_, (2 g) Na_2_HPO_4_, (1.5 g) MgSO_4_·7H_2_O, (1.5 g) yeast extract, (0.1 g) CaCl_2_·2H_2_O, (8 mg) FeCl_3_·6H_2_O, (1 mg) ZnSO_4_·7H_2_O, (0.1 mg) CuSO_4_·5H_2_O, (0.1 mg) Co(NO_3_)_2_·6H_2_O, (0.1 mg) MnSO_4_·5H_2_O with pH adjusted at 6 ± 0.2) was inoculated by 100 µL of fungal spore suspension (10^5^ spores/mL) [29]. At 30 °C for 7 days, the flasks were incubated under both shaking at 120 rpm and static conditions [30].

### Determination of reducing sugars

**Fig. 1:**
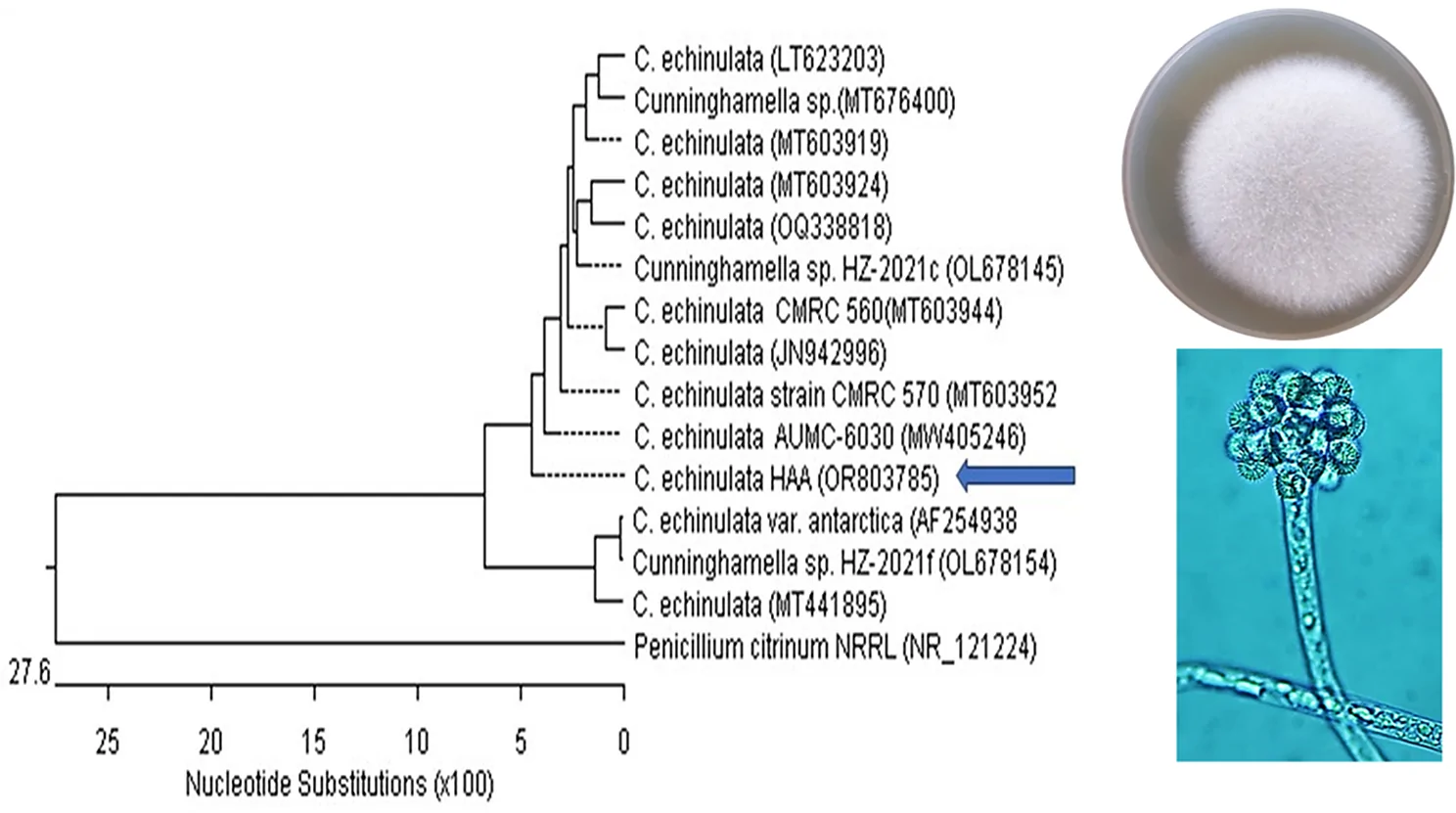
Phylogenetic tree based on ITS gene sequences of *C. echinulata* HAA

After incubation, a reagent of 3,5-dinitrosalicylic acid (DNS) was used to calculate the reducing sugar. One mL of the sample (biomass-free medium) and 2 mL of the DNS reagent were mixed and heated in a boiling water bath for 5 min. The mixture was cooled to room temperature, and 2 mL of distilled water was added. The absorbance was measured using a spectrophotometer at 570 nm to quantify sample’s sugar content which calculated using D-glucose standard curve (Fig. 1S) [31]. The sugar was calculated by comparing the total sugar in the production medium before incubation to the available sugar after incubation.

### Cell dry weight determination and total lipids quantification

**Fig. 2:**
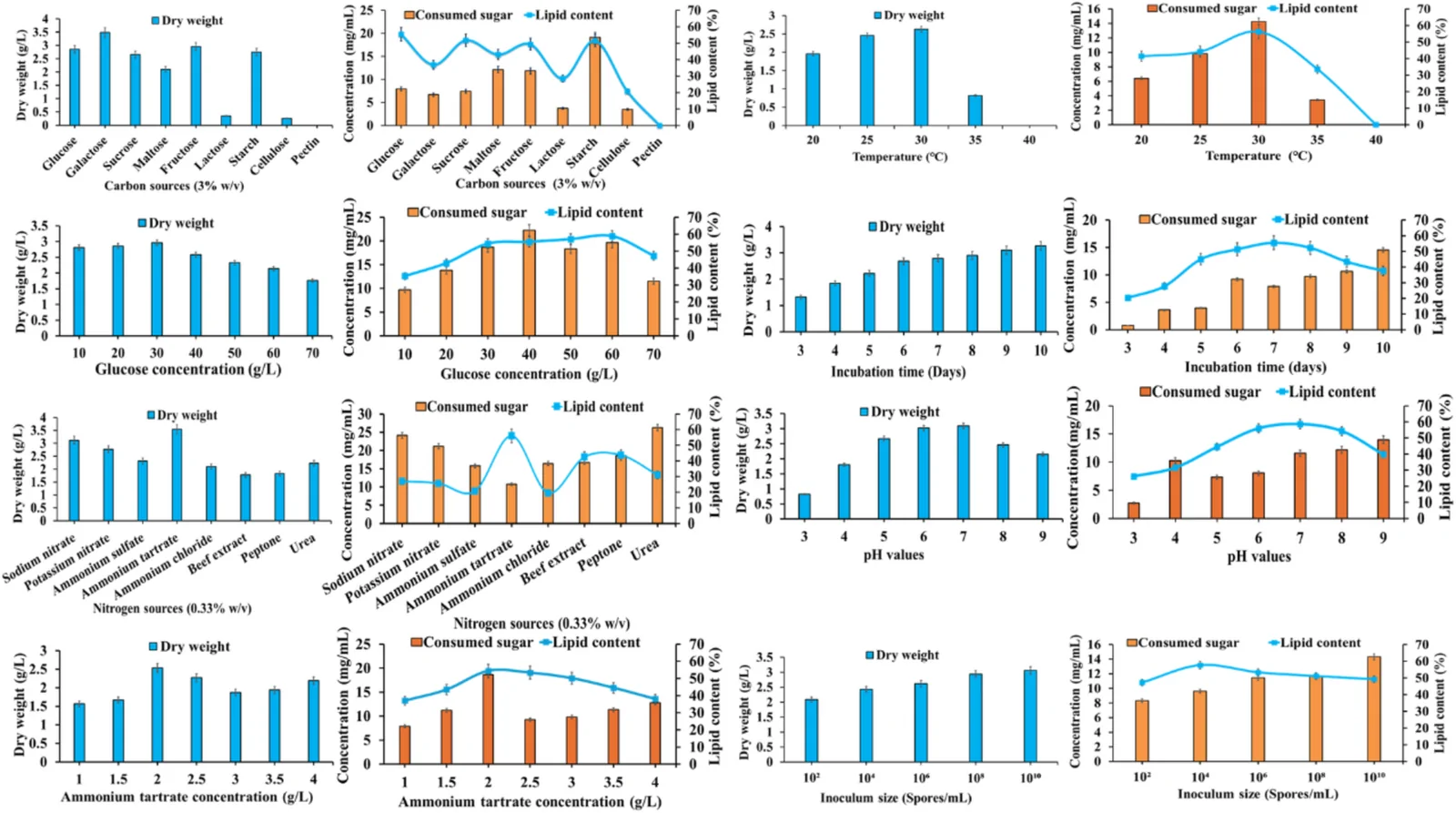
Environmental and nutritional conditions affecting growth and lipid content by *C. echinulata*

The fungal biomass was collected by filtration, and then dried at 55 °C in an oven until a constant weight. One gram of grounded biomass was combined with 40 mL (2:1) chloroform-methanol on orbital shaker for 20 min at 20 °C, filtered with Whatman paper no. 1, and mixed 0.9% NaCl solution. The lipid-containing lower layer was separated, allowed to evaporate, and determination of lipid yield using the sulfo-phospho vanillin (SPV) method was carried out [32]. The sample was mixed with 2 mL of sulfuric acid (98% conc.), heated at 100 °C for 10 min, cooled for 5 min in an ice bath, and then combined with 5 mL of freshly prepared SPV (stirring 0.6 g of vanillin in 10 mL of ethanol (10%) followed by 400 mL of concentrated phosphoric acid). For 15 min at 37 °C, the mixture was incubated under 200 rpm shaking. Using spectrophotometer (Janeway 7315) at 530 nm, absorbance was measured to quantify sample’s lipid content in comparison to of canola oil as a reference standard (Fig. 2S) [20].

### Optimization of culture conditions to maximize lipid biosynthesis

#### Effect of different carbon sources

The effects of different carbon sources were investigated using 250-mL shaken flasks containing 100 mL of broth medium. Nine different carbon sources, including glucose, galactose, fructose, lactose, maltose, sucrose, starch, cellulose, and pectin, were tested for their impact on growth and lipid biosynthesis. Each carbon source was added separately to the medium at a concentration of 3% (w/v). *C. echinulata* was then inoculated into the respective medium and incubated at 30 °C for 7 days at 120 rpm on a shaker incubator [33]. Following the optimal incubation period, lipid content, consumed sugar, and biomass production were quantified. After identifying the best carbon source, the optimal concentration (10, 20, 30, 40, 50, 60, and 70 g/L) of the selected carbon source was also determined.

#### Effect of different nitrogen sources

Organic nitrogen sources (peptone, beef extract, and urea) and inorganic nitrogen sources (sodium nitrate, ammonium sulfate, ammonium tartrate, ammonium chloride, and potassium nitrate} were added separately to the media at a concentration of 0.33% (w/v). The isolate was inoculated into the respective medium and then incubated at 30 °C for 7 days at 120 rpm on a shaker incubator. After that, the lipid content, consumed sugar, and biomass production were quantified. After identifying the best nitrogen source, the optimal concentration (1, 1.5, 2, 2.5, 3, 3.5, and 4 g/L) of the selected nitrogen source was also determined [34].

#### Effect of different incubation temperatures

The study investigated the impact of different incubation temperatures (20, 25, 30, 35, and 40 ℃) on growth and lipid biosynthesis. This was done by inoculating 1 mL of the spore suspension of a selected isolate into the production medium and then placing it in a shaker incubator at 120 rpm for 7 days [33]. Growth, lipid content, and sugar consumption were determined.

#### Effect of different pH values

The production medium was initially adjusted at different pH values of 3, 4, 5, 6, 7, 8, and 9 using 1 N HCl and 1 N NaOH. All pH adjustments were made before sterilizing the medium. Each flask contained 100 mL of the medium. After autoclaving, each flask was inoculated with fungal spores and incubated on a shaker incubator at 120 rpm for 7 days at 30º C [33]. Following incubation, analyses were conducted to determine lipid content, sugar consumption, and biomass.

#### Effect of different incubation times

To determine the optimum incubation period, fermentation flasks were incubated for various durations (3, 4, 5, 6, 7, 8, 9, 10 days). Lipid content was analyzed every 24 h, starting on the third day of the incubation period [33].

#### Effect of different inoculum sizes

To determine the optimum inoculum size, fermentation flasks were inoculated with different concentrations of spores (10², 10⁴, 10⁶, 10⁸, and 10¹⁰ spores/mL). The flasks were incubated on a shaker incubator at 120 rpm for 7 days at 30 °C [35]. Following incubation, analyses were conducted to determine lipid content, sugar consumption, and biomass.

### Optimization of lipid biosynthesis using statistical design

Response surface methodology (RSM) utilizing a Box–Behnken design (BBD) was implemented to identify the optimal factors for lipid biosynthesis by *C. echinulata*. The studied optimization factors/variables were presented in Table 1.

**Table 1:** Independent factors levels of Box–Behnken design

No.	Symbol	Variables	Units	Range		
				Minimum	Maximum	Mean
1	A	Temperature	°C	25	35	30
2	B	Initial pH	-	6	8	7
3	C	Incubation time	Days	6	8	7
4	D	Inoculum size	Spores/mL	10^2^	10^6^	10^4^
5	E	Glucose concentration	g/L	50	70	60
6	F	Ammonium tartrate concentration	g/L	1.5	2.5	2

A total of fifty-four experiments, including central points, were conducted to model the polynomial relationship based on the BBD with six variables [36]. The software generated the coefficients for the variables and their interactions and contour plots. The optimal values for the studied variables were determined through analysis of the equation regression and construction of response plots. The coefficient of determination (R²) was used to evaluate the polynomial equation’s explanatory power, and employing the F-test to assess significance.

### Effect of different waste substrates on growth and lipid biosynthesis

Ten grams of each grape, peach, apricot, and plum waste were taken into 90 mL of distilled water for mechanical and physical treatment before use as a culture medium. Mechanical treatment involved using a 1000 rpm mechanical electrical hand blender for 5 min, followed by physical treatment through boiling for 20 min. The mixture was then centrifuged at 6000 rpm for 15 min. After centrifugation, the supernatant (juice) was transferred to a 250 mL flask and diluted to 100 mL with distilled water. Molasses was dissolved in distilled water (10 g of each in 90 mL). The pH has been adjusted at 7 ± 0.2. The supernatants and diluted molasses were used as media without any extra nutrients for the growth of *C. echinulata*. Finally, reducing sugar was determined for each diluted hydrolysate before sterilization [22]. Following incubation, analyses were conducted to determine lipid content, sugar consumption, and biomass.

### GC-MS analysis

The fatty acid profile of highest extracted lipid contents from *C. echinulata* cultivated in basal medium, OFAT, BBD, and wastes were performed to detect saturated and unsaturated fatty acids. Sulfuric acid was used as a catalyst in flasks to perform the trans-esterification reactions under the following circumstances: 30:1 methanol to oil molar ratio, 160 rpm, 55 °C temperature, 5 h reaction time, and 80% catalyst quantity based on oil weight [37]. In a separating funnel, the reaction mixture was cooled until two layers formed. Chloroform was used to remove the top layer, which includes fatty acid methyl esters (FAMEs). Chloroform was then evaporated from the solution to yield the final FAME products. GC-MS was used to analyze and detect FAMEs [5]. Principal component analysis (PCA) was used to find characteristics that account for the majority of the variability and to choose the best indicators (a minimum data set, or MDS) that represent the influence on lipid content of fatty acids detected by GC-MS and produced through basal medium, optimized factors, statistical, and fruit waste.

### Assessment of the antimicrobial activity of extracted lipids

The antimicrobial potential of highest extracted lipid contents from *C. echinulata* cultivated in basal medium, OFAT, BBD, and wastes was evaluated against pathogenic bacteria: *Staphylococcus aureus* ATCC 6538, *Bacillus subtilis* ATCC 6633, and *Salmonella typhimurium* ATCC 14,028 and *Escherichia coli* ATCC 8739 in addition to yeasts: *Candida albicans* MLBM 73 and *C. glabrata* C-4, and molds: *Aspergillus flavus* TU-61 and *A. niger* ATCC 16,404. At 37 °C for 24 h, bacterial strains were grown in nutrient agar (NA), while filamentous fungi were grown for 5 days on PDA medium at 28 °C, and *Candida* spp. were grown for 2 days at 25 °C on Sabouraud dextrose agar medium (SDA).

The well diffusion technique was used to assess the extracted lipids’ antimicrobial properties as described by Sayegh et al. [38]. One hundred mg of lipids were dissolved in 0.9 mL of 10% dimethyl sulfoxide (DMSO) (Millipore Sigma—St. Louis, MI, USA) to obtain a final concentration of 100 mg/mL. NA, SDA, and PDA plates, were inoculated separately with bacterial, *Candida* spp., and fungal strains, respectively. 50 µL of the test lipid stock was added to wells (diameter ~ 6 mm). Plates were refrigerated until the substance was saturated into the wells, after which they were incubated at 37 °C for 24 h, 28 °C for 3–5 days, and 28 °C for 2 days for bacteria, fungi, and Candida, respectively. The inhibitory zones’ diameter was then measured in millimeters (mm). Chloramphenicol (1 mg/mL) and fluconazole (1 mg/mL) were utilized as positive controls for bacteria and fungus, respectively, while DMSO (10%) served as a negative control. Every test was run three times. A dilution series (100, 50, 25, 12.5, and 6.25 mg/mL) was used to determine the minimum inhibitory concentration (MIC) for positive activities. The MIC was established as the lowest lipid concentration that totally inhibits the pathogen’s growth [39].

### Statistical analysis

Every experiment was run three times. The data of optimization of culture conditions and effect of different waste substrates on lipid biosynthesis were reported as the mean ± standard error (SE) and determined to be below the 0.05 level of significance using multiple comparison tests (Duncan) and analysis of variance (one-way ANOVA) using the SPSS program, version 16 (IBM, Armonk, NY, USA). The BBD for statistical optimization was done by using Minitab 19^®^ software (Version 19.1.1.0, LLC). SPSS software was used to perform PCA, while Origin 2018 software (USA, Origin Lab) was used to create graphical presentation.

## Results

*Cunninghamella echinulata* produced lipid yield of 55.37% in basal medium under shaking.

### Molecular identification of *C. echinulata*

**Fig. 3:**
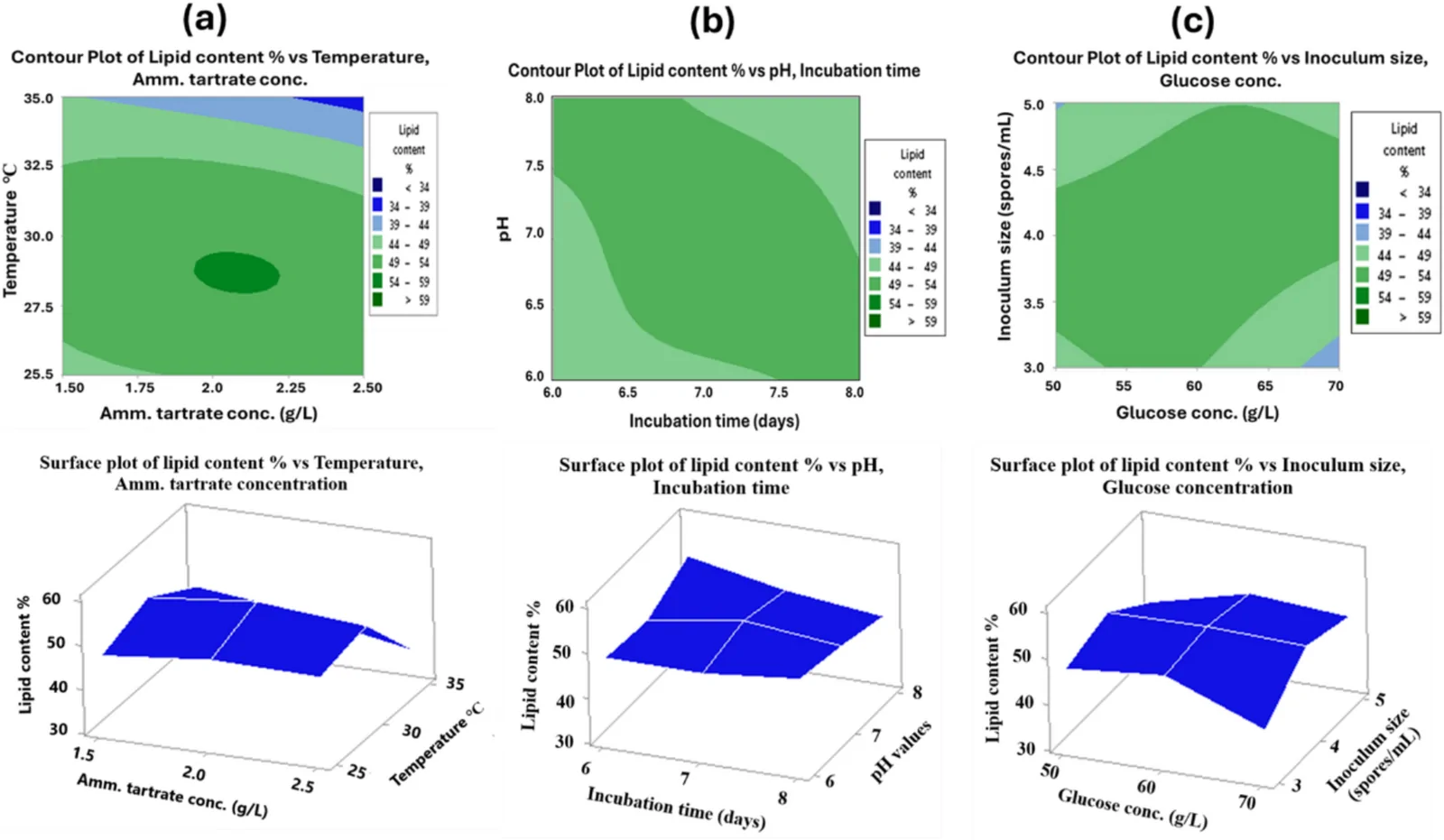
Contour and surface plots show the significant effects of the investigated factors and their interactions on lipid content of *C. echinulata*

Molecular identification had distinguished the fungal isolate from strains of the same species. The fungal isolate’s obtained sequence (386 base pairs) was compared to the GenBank database, and BLAST revealed that the sequence was 98–100% identical to recorded sequences of *C. echinulata*. Accordingly, the fungal isolate was named *C. echinulata* isolate HAA, and the obtained sequence was deposited in GenBank with accession number (OR803785). The phylogenetic tree and color-coded heat map were constructed from the aligned sequences, showing that the strains were clustered into one species (Figs. 1 and 3S).

### Effects of environmental and nutritional OFAT conditions on growth and lipid biosynthesis by *C. echinulata*

Regarding carbon sources, galactose promoted the highest growth of *C. echinulata* (3.49 g/L) by consuming 22.4% of the total sugar, while fructose yielded the second-highest growth (2.96 g/L) by consuming 39.5% of the total sugar. Glucose, starch, and sucrose produced 2.86, 2.75, and 2.66 g/L of dry cell biomass, consuming 26.5%, 63.87%, and 24.77% of the total sugar, respectively. Pectin was unfavorable for the growth and lipid biosynthesis by *C. echinulata*. Glucose was assessed as the best source of carbon for lipid accumulating 55.45% of dry biomass, followed by sucrose and starch with 51.79% and 51.55%, respectively. Growth and lipid content decreased when lactose and cellulose were utilized, resulting in 0.35 and 0.26 g/L dry weight and 28.57% and 20.80% lipid content, respectively (Fig. 2).When various concentrations of glucose, ranging from 10 to 70 g/L, were tested, the most effective concentration for lipid biosynthesis was 60 g/L of glucose, which yielded a lipid content of 58.84%, and consumed 32.75% of the total glucose (Fig. 2).

Ammonium tartrate, as a nitrogen source, was the most effective nitrogen source for maximizing mycelial growth and lipid accumulation in *C. echinulata*. The obtained values were 3.55 g/L dry biomass weight and 56.04% lipid content, with the lowest glucose consumption (35.77%). On the other hand, using ammonium chloride resulted in a significant decrease in growth (2.1 g/L) and lipid content (19.53%). Moreover, beef extract and peptone reduced mycelial dry weight to 1.78 g/L and 1.83 g/L, respectively (Fig. 2). Different concentrations of ammonium tartrate, ranging from 1 to 4 g/L, showed that 2 g/L of ammonium tartrate was the most effective, yielding the highest biomass at 2.54 g/L and a lipid content of 54.47%, consuming 62.5% of the total sugar. Both high and low concentrations of ammonium tartrate (4 and 1 g/L) significantly reduced fungal growth and lipid content (Fig. 2).

The fungus demonstrated significant growth between 20 and 35 °C but could not grow at 40 °C. Maximum growth occurred at 30 °C, producing the highest biomass at 2.63 g/L and maximum lipid content of 56.27%. Glucose consumption significantly increased from 21.37% to 47.6% as the temperature increased from 20 to 30 °C but decreased at high temperatures (Fig. 2).

The results indicated that the optimal pH for both growth and lipid biosynthesis by *C. echinulata* was pH 7.0, with a dry biomass yield of 3.09 g/L, and a lipid content of 58.60%, with 38.57% of total sugar utilized. In contrast, the lowest fungal biomass and lipid yield were observed at pH 3.0, with significantly reduced values of 0.82 g/L of dry biomass and 26.20% lipid content (Fig. 2).

The results indicated that extending the incubation period from three to ten days significantly increased growth, with biomass raised from 1.32 to 3.27 g/L and increased sugar consumption. The optimal incubation time for lipid accumulation was seven days, yielding 55.5% lipid content. Shorter and longer incubation periods significantly decreased lipid content (Fig. 2).

The inoculum size of C. *echinulata* (10^4^ spores/mL) resulted in the highest lipid yield of 57.70%. Meanwhile, the maximum biomass production (3.06 g/L dry weight) was achieved with an inoculum size of 10^10^ spores/mL. Additionally, glucose consumption increased with a high value of inoculum size. Conversely, the lowest biomass and lipid content were recorded at the minimum inoculum size of 10^2^ spores/mL; yielding only 2.09 g/L dry weight and 47.23% lipid content (Fig. 2).

This study found that (60 g/L) of glucose and (2 g/L) of ammonium tartrate were the optimal nutritional factors for achieving the highest lipid production by *C. echinulata*. The best results were obtained at 30 °C, over 7 days, using a spore concentration of 10⁴ spores/mL and maintaining a pH of 7, which increased the lipid content to 61.23% compared to the original fermentation medium (55.37%). The C/N ratio for high lipid content was 78.4.

### Optimizing lipid biosynthesis by RSM-BBD

Table 2 showed the independent factors, and their respective levels employed in optimizing lipid biosynthesis. It illustrated the Box–Behnken design of these factors and the predicted and experimental values. The lipid biosynthesis by *C. echinulata* was predicted using the following equation:

$$\begin{aligned}\text{Lipid content } (\%)&=-64.43 + 1.861A + 3.41B\\&\quad+ 5.20C-0.800D + 0.370E\\&\quad+ 5.58F- 0.02660A^{2}\\&\quad- 0.1594B^{2} - 0.2465C^{2}\\&\quad- 0.1024D^{2} - 0.002669E^{2}\\&\quad- 0.592F^{2}- 0.0046AB\\&\quad- 0.0041AC + 0.00569AD\\&\quad - 0.00228AE- 0.0759AF\\&\quad - 0.1540BC + 0.0943BD\\&\quad - 0.00079BE - 0.147BF\\&\quad - 0.0161CD - 0.00930CE\\&\quad + 0.0396CF\end{aligned}$$

The highest lipid content of *C. echinulata* (59.37%) was achieved in run no. 47, while the lowest lipid content (34.61%) was observed in run No. 13. The conditions for run no. 47 included 30 °C incubation temperature, pH value of 7 for 7 days incubation time, inoculum size of 10⁴ spores/mL with 60 g/L and 2 g/L of glucose and ammonium tartrate concentrations, respectively.

Analysis of variance (ANOVA) for the quadratic model of lipid accumulation in *C. echinulata* was presented in Table 3. At a *p*-value < 0.05, significant model terms with were considered. The model’s F-value of 9.88 confirmed its significance. One-way ANOVA revealed that temperature significantly affected lipid biosynthesis, while the other factors were non-significant. Conversely, two-way ANOVA showed that interactions between temperature with ammonium tartrate concentration, pH with incubation time, and inoculum size with glucose concentration were significant for lipid biosynthesis, while other interactions were non-significant.

The “Lack of Fit F-value” of 28.96 suggested that the lack of fit was significant compared to pure error. The pure error could be calculated depending on repeats of specific design points such as central points represented by runs no. 21, 29, 42, 44, 47, and 54. The resulting multiple correlation coefficient (R² = 0.9112) was closing to 1, indicating a preferable interconnection correlation between predicted and experimental and values, demonstrating the model’s accuracy in predicting the response.

**Fig. 4:**
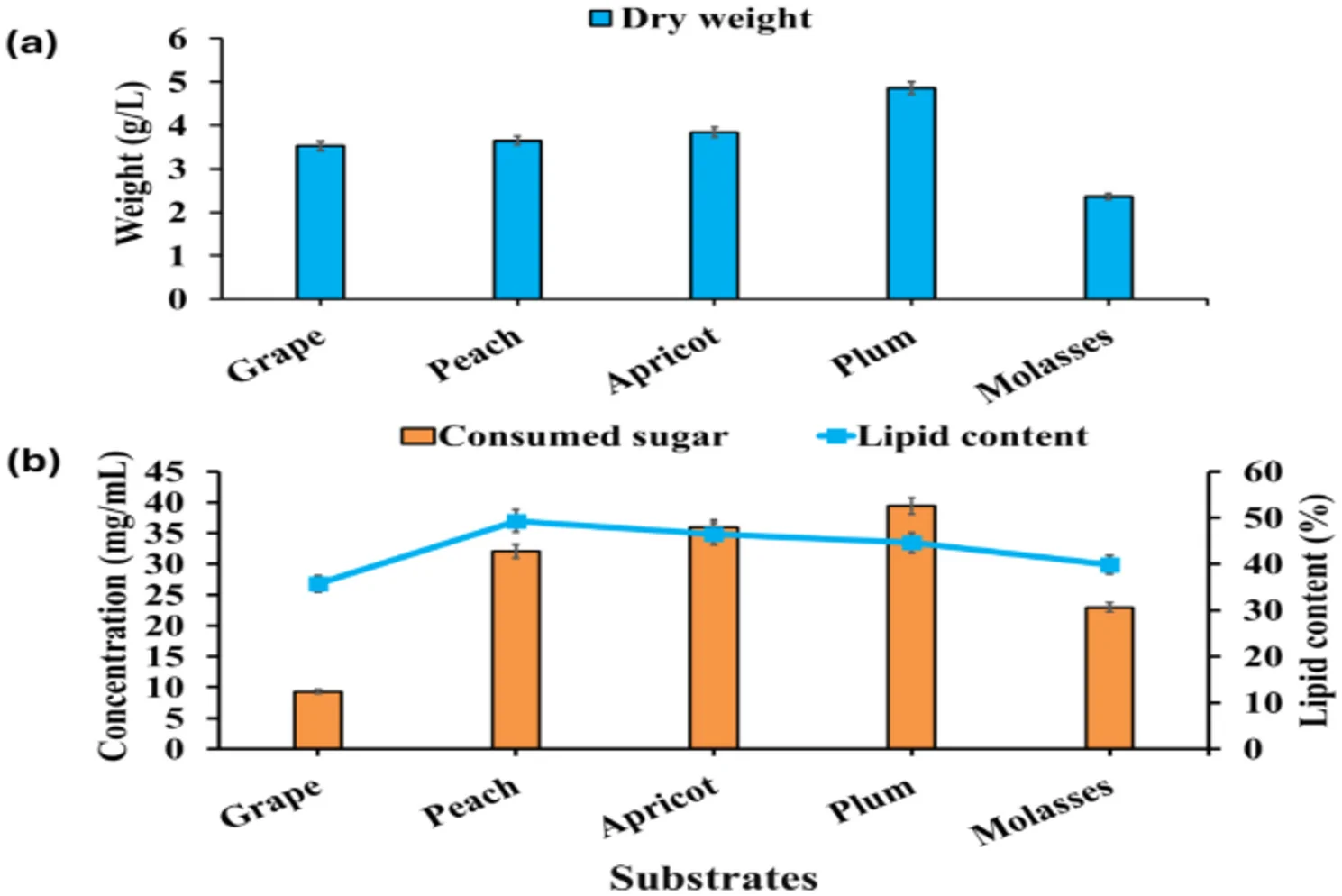
Effect of different wastes on (**a**) growth and (**b**) lipid content of *C. echinulata*

Pareto chart and normal probability plot (Fig. 4S), contour and surface plots (Fig. 3a, b, and c) further illustrated the relationship between parameters and defined the optimal conditions for lipid biosynthesis by *C. echinulata*.

**Table 2:** Optimization variables using Box–Behnken design showing experimental and predicted lipid content of *C. echinulata*

Run order	Variables						Lipid content (%)	
	A	B	C	D	E	F	Experimental response	Predicted response
1	30	8	8	10^4^	50	2	48.88	49.16
2	30	6	7	10^4^	50	1.5	51.59	50.71
3	25	7	7	10^2^	70	2	46.61	47.26
4	30	8	7	10^4^	50	1.5	50.65	53.17
5	30	8	7	10^4^	50	2.5	49.93	49.57
6	35	7	7	10^6^	50	2	40.9	40.29
7	25	7	6	10^4^	60	1.5	44.98	45.95
8	30	7	8	10^6^	60	1.5	52.61	52.88
9	35	7	8	10^4^	60	2.5	37.47	37.38
10	25	7	7	10^6^	50	2	44.95	44.56
11	30	6	6	10^4^	50	2	48.27	46.13
12	30	8	6	10^4^	50	2	50.85	50.75
13	35	7	7	10^2^	70	2	34.61	35.71
14	30	6	8	10^4^	50	2	52.97	53.22
15	30	6	8	10^4^	70	2	49.82	49.85
16	25	7	8	10^4^	60	1.5	48.96	46.09
17	30	6	7	10^4^	50	2.5	51.12	51.32
18	30	7	8	10^6^	60	2.5	50.35	50.77
19	35	6	7	10^6^	60	2	40.58	41.3
20	30	7	8	10^2^	60	2.5	56.5	52.73
21	30	7	7	10^4^	60	2	59.16	59.23
22	35	7	6	10^4^	60	1.5	44.78	43.38
23	25	8	7	10^2^	60	2	49.19	49.37
24	35	7	7	10^6^	70	2	42.24	41.49
25	35	8	7	10^6^	60	2	43.62	43.26
26	30	6	7	10^4^	70	1.5	49.14	49.41
27	35	8	7	10^2^	60	2	40.61	39.83
28	30	7	6	10^6^	60	2.5	49.97	50.52
29	30	7	7	10^4^	60	2	59.16	59.23
30	25	6	7	10^2^	60	2	48.07	51.28
31	25	7	8	10^4^	60	2.5	49.52	51.08
32	30	7	6	10^6^	60	1.5	51.83	53.79
33	35	7	8	10^4^	60	1.5	42.8	42.45
34	30	7	6	10^2^	60	2.5	51.43	51.55
35	25	7	7	10^2^	50	2	50.67	50.38
36	25	8	7	10^6^	60	2	54.29	51.54
37	30	8	7	10^4^	70	2.5	47.27	49.04
38	30	6	6	10^4^	70	2	48.59	48.01
39	25	6	7	10^6^	60	2	47.79	48.12
40	30	8	6	10^4^	70	2	54.28	52.26
41	30	6	7	10^4^	70	2.5	55.44	51.23
42	30	7	7	10^4^	60	2	59.28	59.23
43	35	6	7	10^2^	60	2	42.09	42.73
44	30	7	7	10^4^	60	2	59.14	59.23
45	35	7	6	10^4^	60	2.5	35.64	37.28
46	25	7	7	10^6^	70	2	52.5	52.21
47	30	7	7	10^4^	60	2	59.37	59.23
48	30	7	8	10^2^	60	1.5	51.11	51.23
49	25	7	6	10^4^	60	2.5	49.52	49.81
50	30	8	7	10^4^	70	1.5	51.37	51.38
51	30	8	8	10^4^	70	2	44.83	45.49
52	30	7	6	10^2^	60	1.5	52.22	51.2
53	35	7	7	10^2^	50	2	44.15	44.31
54	30	7	7	10^4^	60	2	58.98	59.23

A: Incubation temperatures, B: pH value, C: incubation times, D: inoculum sizes, E: glucose concentrations, and F: ammonium tartrate concentrations

**Table 3:** ANOVA for the experimental results of lipid content of *C. echinulata*

Source	Sum of squares	Degree of freedom	Mean of squares	F value	*p* value
Model	9.1757	27	0.33984	9.88	0.000
Linear	2.3059	6	0.38432	11.17	0.000
A	2.2546	1	2.25458	65.52	0.000
B	0.0004	1	0.00040	0.01	0.915
C	0.0005	1	0.00054	0.02	0.901
D	0.0027	1	0.00267	0.08	0.783
E	0.0252	1	0.02523	0.73	0.400
F	0.0225	1	0.02251	0.65	0.426
Square	5.7396	6	0.95660	27.80	0.000
A2	3.9132	1	4.54821	132.18	0.000
B2	0.2806	1	0.26135	7.60	0.011
C2	0.6589	1	0.62517	18.17	0.000
D2	0.0272	1	0.10780	3.13	0.088
E2	0.6344	1	0.73293	21.30	0.000
F2	0.2253	1	0.22531	6.55	0.017
2-Way Interaction	1.1302	15	0.07534	2.19	0.039
AB	0.0042	1	0.00419	0.12	0.730
AC	0.0033	1	0.00333	0.10	0.758
AD	0.0129	1	0.01293	0.38	0.545
AE	0.1044	1	0.10440	3.03	0.093
AF	0.2882	1	0.28816	8.37	0.008
BC	0.1896	1	0.18962	5.51	0.027
BD	0.0711	1	0.07112	2.07	0.162
BE	0.0010	1	0.00100	0.03	0.866
BF	0.0431	1	0.04305	1.25	0.274
CD	0.0021	1	0.00207	0.06	0.808
CE	0.0691	1	0.06913	2.01	0.168
CF	0.0063	1	0.00629	0.18	0.673
DE	0.2999	1	0.29986	8.71	0.007
DF	0.0314	1	0.03138	0.91	0.348
EF	0.0036	1	0.00364	0.11	0.748
Residual	0.8946	26	0.03441		
Lack-of-Fit	0.8873	21	0.04225		
Pure Error	0.0073	5	0.00146	28.96	0.001
Total	10.0703	53			

R^2^: 0.9112, adjusted R^2^: 0.8189, and predicted R^2^: 0.5386

### Effect of different wastes as substrates for growth and lipid biosynthesis by *C. echinulata*

The results showed that apricot waste hydrolysate had the highest sugar content of 53.86 mg/mL, while grape waste hydrolysate had the lowest at 23.65 mg/mL. Plum demonstrated a high growth rate with a fungal biomass dry weight of 4.86 g/L, utilizing 85.38% of the total sugar. In contrast, molasses produced a lower fungal growth rate of 3.53 g/L and consumed 39.41% of the total sugar.

The results found that peach waste hydrolysate was the most suitable waste for lipid biosynthesis, yielding 49.30% lipid content. This was followed by apricot and plum waste hydrolysates, with 46.54% and 44.57% lipid contents, respectively. The lowest lipid yield of 35.68% was observed with cultivation on grape waste hydrolysate (Fig. 4a and b).

### GC–MS analysis

**Fig. 5:**
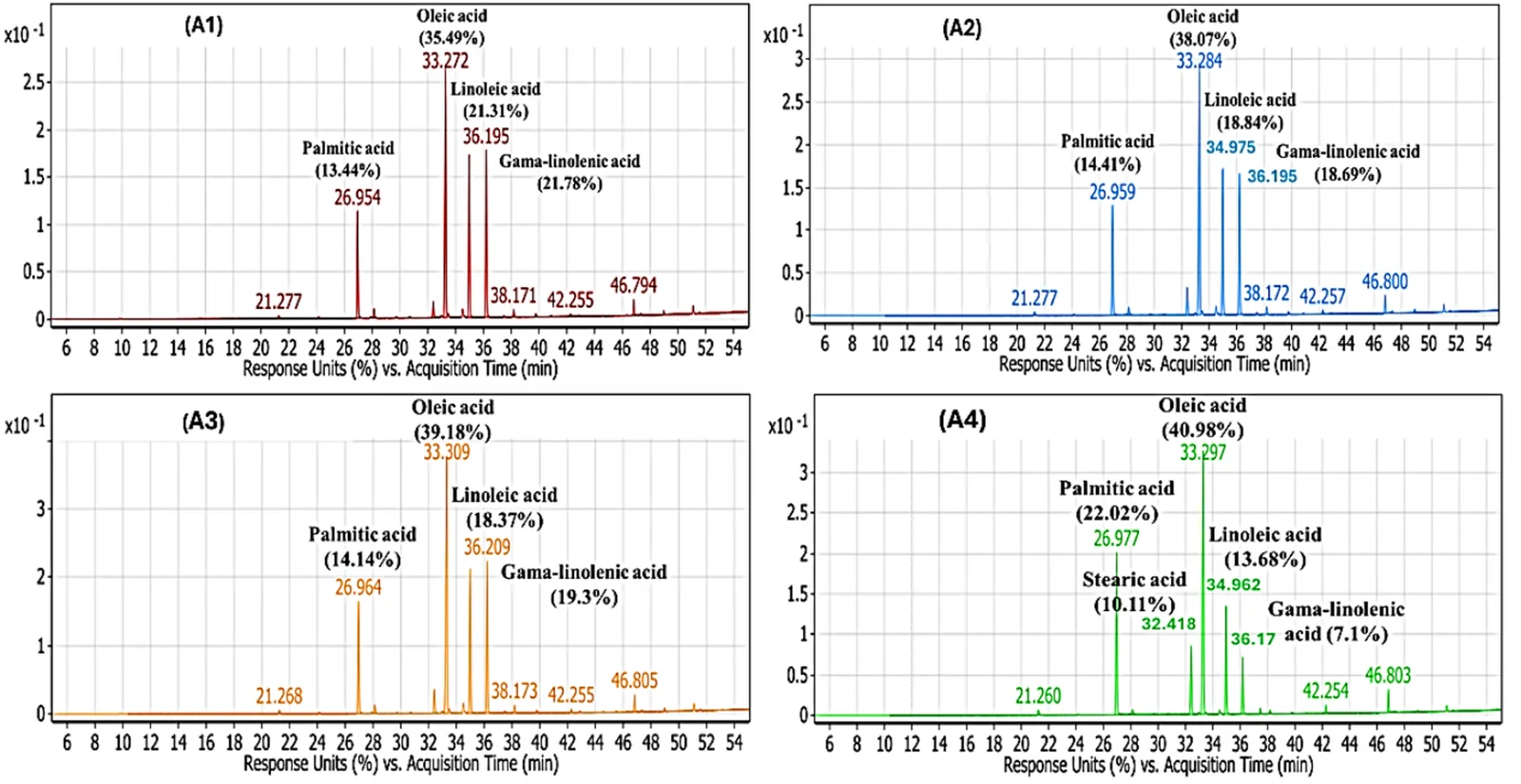
GC-MS chromatogram of the saponifiable lipid extracts (**A1**, **A2**, **A3**, and **A4**) from *C. echinulata*

The results presented in Table 1 S detailed the FAs composition of lipids extracted from the dried mycelium of *C. echinulata* under four different conditions: the basal medium (A1), the OFAT optimized medium (A2), the statistically optimized medium (A3), and the peach waste medium (A4), using GC-MS analysis. Nineteen different FAs were identified (Fig. 5 and S). The lipid extracts contained a mixture of saturated, monounsaturated, and polyunsaturated fatty acids. The most abundant fatty acids were oleic acid, linoleic acid, gamma-linolenic acid, and palmitic acid, which accounted for more than 83% of the total lipid composition in all samples.

Oleic acid, a monounsaturated fatty acid, was present at high percentages: 35.49% in A1, 38.07% in A2, 39.18% in A3, and 40.98% in A4 lipid extracts, suggesting its prevalence across the samples. Gamma-linolenic acid, a polyunsaturated fatty acid, was detected at high percentages in samples A1, A2, and A3, at 21.78%, 18.69%, and 19.3%, respectively, while it was detected at a lower percentage (7.1%) in sample A4. This was followed by the essential polyunsaturated omega-6 linoleic acid that was detected at 21.31%, 18.84%, 18.37%, and 13.68% in samples A1, A2, A3, and A4, respectively. Palmitic acid, a saturated fatty acid, was found at a high percentage of 22.02% in samples A4, while it was detected at low percentages of 13.44%, 14.41%, and 14.14% in samples A1, A2, and A3, respectively. On the other hand, stearic acid was detected at 2.02%, 3.65%, 3.12%, and 10.11% in samples A1, A2, A3, and A4, respectively. Additionally, the saturated lignoceric acid and omega-7 monounsaturated palmitoleic acid were detected in very low amounts, while other fatty acids were present in trace amounts.

**Fig. 6:**
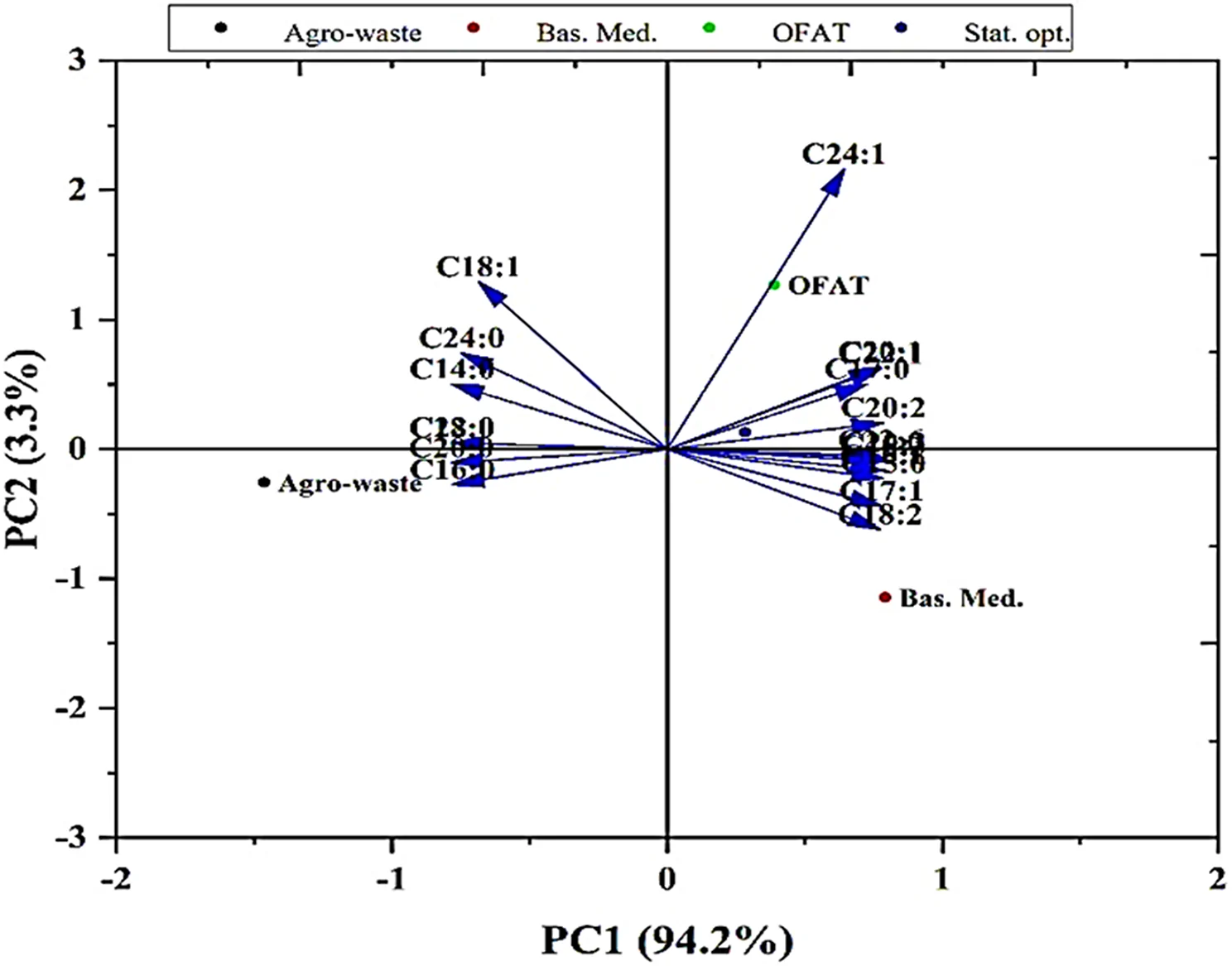
The principal component analysis of fatty acids detected in different lipid extracts of *C. echinulata*

According to the principal component analysis (PCA) data, PC1 is a very significant factor, explaining a significant amount of the variation in analyzed lipids’ FAs using GC-MS produced through basal, optimized OFAT, statistical optimized, and peach waste medium. The first factor accounted for 94.2%, and the second was 3.3%. As demonstrated by the clustering in Fig. 6, basal medium, optimized factor medium, and statistical optimization of the lipids were strongly correlated with C16:1, C17:0, C17:1, C18:1, C18:2, C18:3, C20:1, C20:2, C20:3, C22:1, C24:1, and C22:6 and were having positive values of PC1; these clusters were explained primarily by the high positive values of PC1.

### Antimicrobial activity assay

The data presented in Table 2 S indicated that all lipid extracts (A1, A2, A3, and A4) exhibited antimicrobial activities against most tested microorganisms, except *S. typhimurium*, *A. niger*, and *A. flavus*. The antimicrobial zones of inhibition ranged from 9 to 19 mm when 50 µL of 10% lipid extracts were used. Lipid extract A3 demonstrated the highest antibacterial effect against *E. coli* with an inhibition zone diameter of 19 mm, followed by A4, A2, and A1 with inhibition zones of 17 mm, 16 mm, and 16 mm, respectively. Lipid extract A4 showed a strong efficacy towards *S. aureus* with 18 mm zone of inhibition, followed by A1, A3, and A2 extracts with 17, 17, and 14 inhibition zone diameters, respectively.

**Fig. 7:**
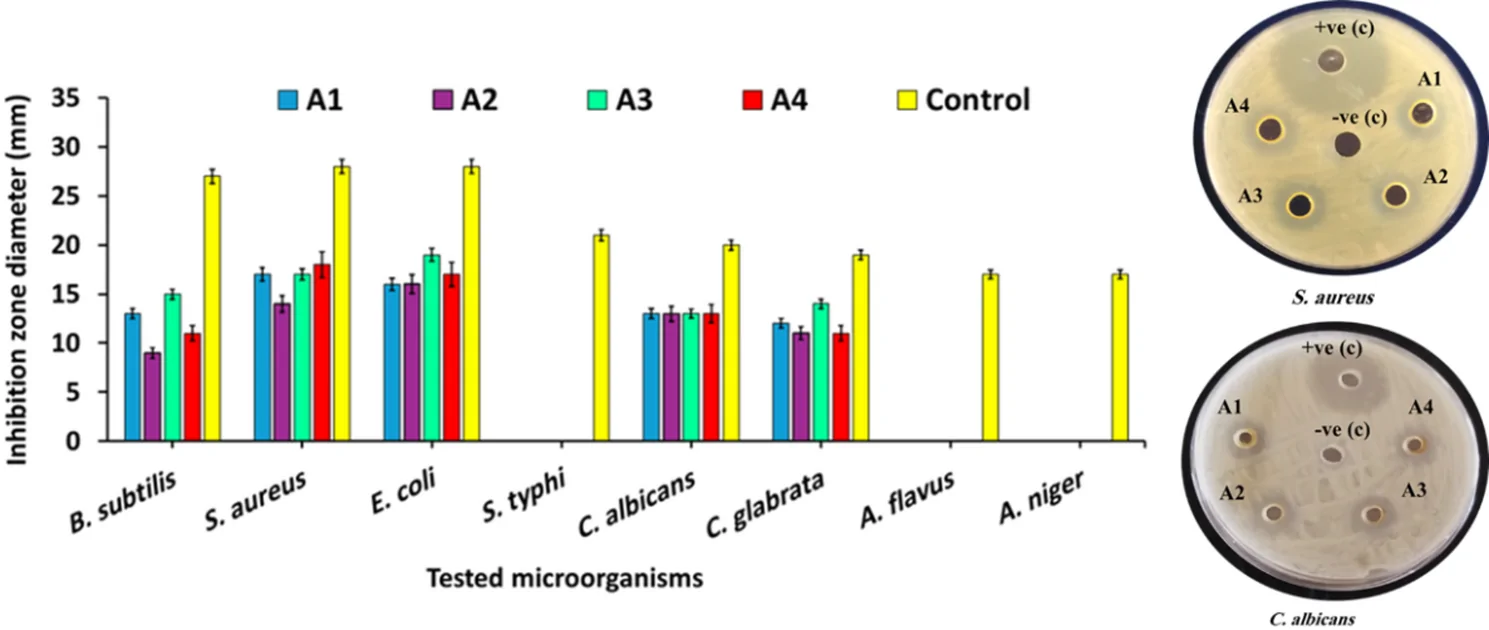
Antimicrobial activity of lipid extracts from *C. echinulata* against tested microorganisms

The activity of all lipid extracts against *B. subtilis* was modest, with zones of inhibition ranging from 9 to 15 mm in diameter. However, *S. typhimurium* showed resistance to all lipid extracts. Additionally, the bioactivity of all extracted lipids demonstrated moderate antifungal activity against *C. albicans* and *C. glabrata*, with inhibition zones ranging from 11 to 14 mm, respectively (Fig. 7). No activity was observed against *A. flavus* and *A. niger*. These results indicated that Gram-positive bacteria (*B. subtilis* and *S. aureus*) were more susceptible to the extracted lipids than Gram-negative bacteria (*E. coli* and *S. typhimurium*), and the lipid extracts were effective against *Candida* species (*C. albicans* and *C. glabrata*), but not against filamentous fungi (*A. flavus* and *A. niger*).

The MICs of the tested lipid extracts (A1, A2, A3, and A4) against bacteria (*B. subtilis*, *S. aureus*, and *E. coli*) and *Candida* species (*C. albicans* and *C. glabrata*) were determined using concentrations ranging from 100 to 6.25 mg/mL. The data in Table 3 S indicated that *E. coli* and *S. aureus* exhibited MIC values between 12.5 and 25 mg/mL, while *B. subtilis* showed MIC values from 25 to 50 mg/mL. On the other hand, the *Candida* species demonstrated MIC values ranging from 25 to 50 mg/mL of lipid extracts.

**Fig. 8:**
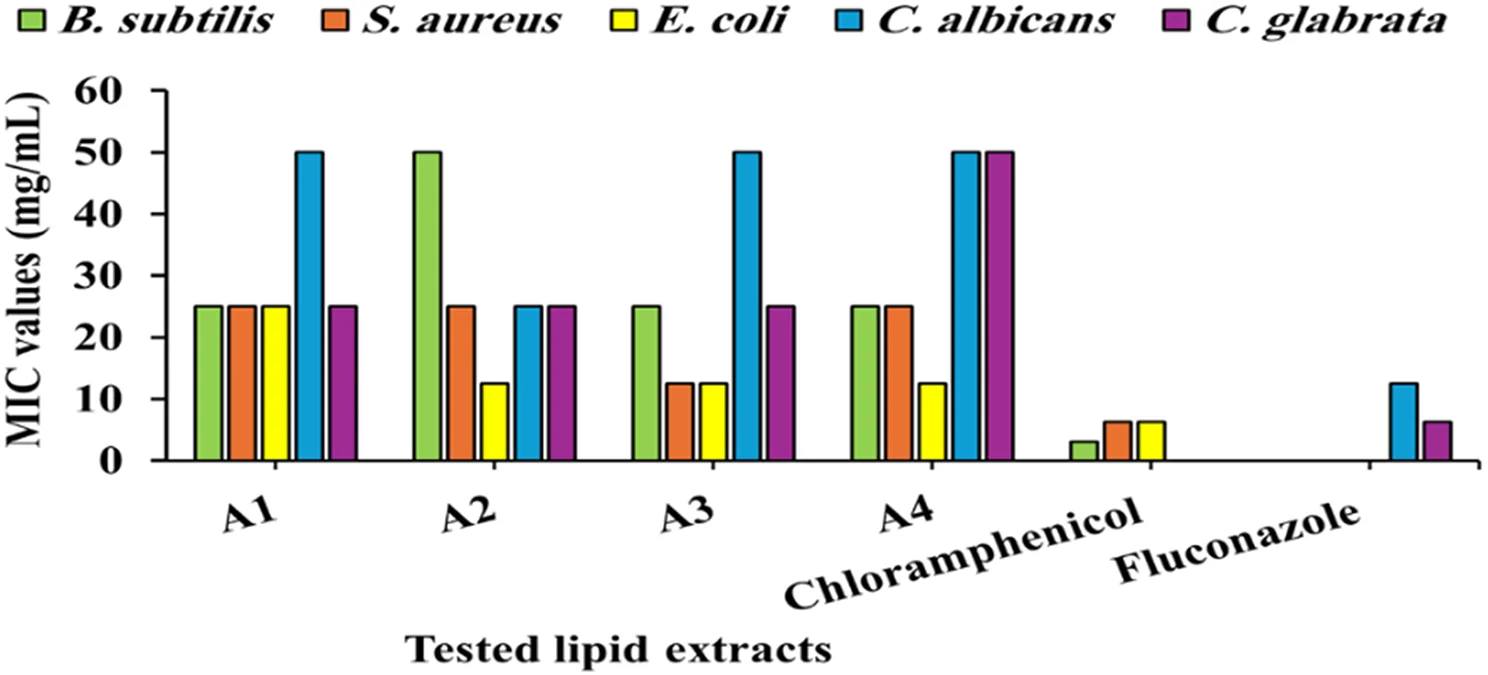
Minimum inhibitory concentration of lipid extracts against tested microorganisms

Lipid extract A1 showed moderate MIC values at 25 mg/mL for *B. subtilis*, *S. aureus*, *E. coli*, and *C. glabrata*, and a higher MIC value at 50 mg/mL for *C. albicans*. Lipid extract A2 recorded a low MIC value at 12.5 mg/mL for *E. coli*, moderate values at 25 mg/mL for *S. aureus*, *C. albicans*, and *C. glabrata*, and a high value at 50 mg/mL for *B. subtilis*. Lipid extract A3 demonstrated low MIC values at 12.5 mg/mL for *S. aureus* and *E. coli*, moderate at 25 mg/mL for *B. subtilis* and *C. glabrata*, and a high value at 50 mg/mL for *C. albicans*. Lipid extract A4 showed a low MIC value at 12.5 mg/mL for *E. coli*, moderate values at 25 mg/mL for *B. subtilis* and *S. aureus*, and high values at 50 mg/mL for *C. albicans* and *C. glabrata* (Fig. 8). Chloramphenicol and Fluconazole showed MIC values at lower concentrations. The MIC values of Chloramphenicol were recorded at 3.125 mg/mL for *B. subtilis*, and 6.25 mg/mL for both *S. aureus*, and *E. coli*, while Fluconazole MIC values were recorded at 12.5 mg/mL for *C. albicans* and 6.25 mg/mL for *C. glabrata*.

## Discussion

Several species belonging to Zygomycetes and Ascomycetes groups are essential for their ability to accumulate significant amounts of lipids in their biomass [28]. Oleaginous microbes can accumulate lipids in their cells at levels exceeding 20% of their dry cell weight because these microbes possess specialized metabolic pathways that direct excess carbon towards lipid biosynthesis, often regulated by the availability of nutrients, particularly nitrogen. When nitrogen is limited, carbon flux is redirected from protein synthesis to lipid accumulation [40].

*Cunninghamella echinulata*, isolated from agricultural soil, exhibited the highest lipid content (55.37%). Do Nascimento et al. [41] isolated *C. echinulata* from soil with a lipid content of 40% w/w, while Firoozabad and Nasr [42] reported a lipid yield of 34.2% by *C. echinulata*. Molecular identification of *C. echinulata* HAA was determined using ITS genetic sequences of 18 S rRNA. Internal transcribed spacer (ITS) sequences have been extensively utilized to identify species within the genus *Cunninghamella* [43]. Hallur et al. [44] highlighted the efficiency of ITS region sequencing as a molecular approach for the valid identification of *Cunninghamella* species.

This comprehensive investigation aimed to optimize the conditions for lipid biosynthesis in *C. echinulata*, contributing to the development of efficient biotechnological processes for industrial applications. *Cunninghamella echinulata*, a potent lipid producer, was subjected to diverse environmental and nutritional factors to study their impact on lipid biosynthesis and growth. Among the most critical factors affecting lipid biosynthesis were incubation temperature, initial pH value, incubation time, and the sources of carbon and nitrogen [45]. The key factors examined included different carbon sources, with glucose being the most effective for the maximum lipid content (55.45%), while ammonium tartrate, as a nitrogen source, was found to maximize both mycelial growth (3.55 g/L) and lipid content (56.04%). Song et al. [46] indicated that glucose is a widely used carbon source for lipid biosynthesis in fungi, including *C. echinulata*. Espinoza Gil et al. [47] observed that low glucose concentrations in the culture medium reduced lipid yields in *C. echinulata*.

However, increasing the glucose concentration enhanced lipid content, reaching a peak at approximately 50 g/L, with lipid synthesis accounting for 39.02% of the biomass. Chatterjee and Mohan [48] examined glucose-based lipid accumulation in fungi, revealing that lipid content can exceed 70% (w/w). Mohamed et al. [28] demonstrated that the highest biomass and lipid biosynthesis were achieved by *M. circinelloides* when using glucose (80 g/L) as the carbon source and ammonium tartrate (2 g/L) as the nitrogen source. Ling et al. [49] showed that glucose and yeast extract were the best for lipid biosynthesis by many genera of Mucorales, such as *Mucor* sp. and *Cunninghamella* sp. Cianchetta et al. [50] reported that *C. echinulata* reached the highest biomass at 19.6 g/L on sucrose compared with other tested sugars. Mohamed et al. [51] found that ammonium tartrate effectively promotes lipid accumulation in *C. echinulata*.

Microorganisms utilize carbon and nitrogen sources to enhance lipid biosynthesis. Glucose is a single sugar unit that can be quickly absorbed and metabolized, leading to efficient energy production and supporting faster growth and lipid synthesis. In contrast, disaccharides and polysaccharides require enzymatic breakdown into monosaccharides before utilization, slowing down the metabolic process. Therefore, monosaccharides are more efficient for immediate energy needs [2]. Similarly, inorganic nitrogen sources such as ammonium and nitrate are directly available for uptake and do not require complex metabolic processes, making them more efficient for growth and lipid synthesis. Conversely, yeast extract as an organic nitrogen sources stimulates high lipid accumulation [52].

The accumulation of lipids by microorganisms depends on the proportion of carbon and nitrogen (C/N ratio) available in the medium, low nitrogen concentration and high carbon concentration lead to high lipid accumulation. The results indicated that the highest lipids were produced by *C. echinulata* at a glucose concentration of 60 g/L and an ammonium tartrate concentration of 2 g/L, with a C/N ratio of 78.4. A recent study found that oleaginous microbes must accumulate the most lipid; the beginning C/N ratio must be higher than 20 [12]. Gema et al. [52] reported that when the nitrogen source does not affect glucose uptake, C⁄N is an important factor affecting lipogenesis. When *C. echinulata* was cultivated on semi-synthetic media, having glucose as the carbon source and ammonium sulfate and yeast extract as nitrogen sources, lipid accumulation was achieved at high C/N [53].

The optimal incubation time for lipid biosynthesis by *C. echinulata* was 7 days, resulting in 55.5% lipid content. Growth and biomass production were significantly influenced by the incubation period, which can result in more lipid accumulation and effective use of the carbon sources in the growth medium [22]. Sukrutha et al. [54] reported that the optimum incubation time for gamma-linolenic acid (GLA) production (21%) by *Cunninghamella blakesleeana* was 6 days.

The ideal temperature for biomass and lipid biosynthesis by *C. echinulata* was 30 °C, accumulating 2.63 g/L dry biomass and 56.27% lipids. Because temperature affects fungal metabolism, enzyme activity, and membrane fluidity, which in turn impact lipid biosynthesis, it was a stress factor that affected oleaginous fungi’s development, lipid accumulation, and fatty acid composition [55]. Umrao et al. [18] reported that low temperature hampers fungal growth and lipid accumulation; moderate temperatures ranging from 25 ℃ to 30 ℃ are favored for growth and lipid accumulation, while high temperatures of 35 ℃ and above impede cell growth and induce lipid accumulation.

The study identified the optimal pH value for *C. echinulata* as 7 for maximizing lipid yield (58.6%) and fungal dry biomass (3.09 g/L). Hashem et al. [22] demonstrated that *C. echinulata* accumulated lipids most effectively at an optimum temperature of 35 °C, with a pH of 7, for high lipid production. Fakas et al. [56] found that the optimal pH values for lipid biosynthesis in fungi, including *C. echinulata*, are typically around neutral pH, ranging from 6 to 7. According to Hashem et al. [20], when the oleaginous fungus *M. racemosus* was cultivated in a rather weak acid medium (pH 5) with a starch content of 20 g/L and an incubation time of 11 days at 30 °C, the maximum lipid (4.28 g/L) was achieved. Lipid accumulation was affected by low and high pH values, as the activity of enzymes involved in lipid biosynthesis was inhibited and the availability of nutrients required for fungal growth was reduced, leading to lower lipid yields [57].

The statistical optimization of lipid biosynthesis by *C. echinulata* using RSM-BBD identified the highest lipid content (59.37%) at 30 °C, pH 7, 7 days of incubation, 10⁴ spores/mL, 60 g/L glucose conc., and 2 g/L ammonium tartrate conc. The analysis revealed that interactions between temperature and ammonium tartrate concentration had an antagonistic effect. Conversely, pH and incubation time synergistically affected lipid content. Similarly, inoculum size and glucose concentration also synergistically affect lipid content.

Optimization studies may be carried out using response surface technique, which is based on a statistical approach [58]. RSM has been widely utilized to assess and comprehend how many important parameters (independent and dependent) interact at various levels to influence microorganisms’ biosynthesis of metabolites [59]. Furthermore, the RSM approach was used to minimize the experiment’s number of trials and determine the optimal parameter with the highest possible outputs [60]. Sekar et al. [61] focused on optimizing biodiesel production from algae oil using RSM, identified the best conditions for catalyst concentration and reaction time to maximize biodiesel yield. Additionally, *Cunninghamella bainieri* was optimized for lipid and gamma-linolenic acid production using RSM, highlighting the significant aeration and agitation levels variables on lipid biosynthesis [62]. Although the model exhibited a high adjusted R² value, the predicted R² was comparatively lower, indicating that the predictive performance of the model was somewhat weaker than its fit to the experimental data. Such differences are not uncommon in response surface methodology studies and may result from experimental variability, unaccounted factors, or the influence of non-significant model terms. However, the predicted R² remained positive and reasonably consistent with the adjusted R², suggesting acceptable predictive ability. Moreover, validation experiments conducted under the optimized conditions yielded lipid content values that closely matched the model predictions, confirming the adequacy and practical reliability of the developed model for optimization of lipid biosynthesis by *C. echinulata*. Moreover, our results showed that the lack of fit was significant compared to pure error. Usually this happens when repeating data are very similar to each other as in the current research. The lack of fit problem is common and reported by many researchers in statistical optimization, and could occur due to noise, and could resulting in “over fitted” model.

However the OFAT and RSM-BBD approaches identified the same optimum conditions for lipid accumulation by *C. echinulata* HAA, the increase obtained by RSM was relatively limited. This result does not necessarily reduce the value of the RSM approach, because OFAT had already identified a culture region close to the maximum lipid-producing capacity of the strain under the tested conditions. Therefore, only a marginal increase was expected when the same variables were further optimized statistically. The practical advantage of RSM in the present study was mainly its ability to validate the optimum conditions, reduce experimental uncertainty, and evaluate the influence and interaction of multiple factors simultaneously. Unlike OFAT, which changes one variable while keeping the others constant, RSM revealed statistically significant interactions between temperature and ammonium tartrate concentration, pH and incubation time, and inoculum size and glucose concentration. These interactions are biologically relevant because lipid accumulation in oleaginous fungi is controlled by the combined effects of carbon availability, nitrogen limitation, growth phase, pH, and temperature-dependent enzyme activity. Recent reviews have emphasized that oleaginous fungi accumulate lipids when carbon is available in excess while nitrogen becomes limiting, redirecting central carbon metabolism toward fatty acid biosynthesis and storage lipid formation [55, 63].

The production of essential compounds from agro-wastes as cheap carbon sources serves as substrates for obtaining highly active compounds used in many industrial and pharmaceutical applications [64]. The present study used rotten fruits and molasses to cultivate *C. echinulata* for lipid production, and results indicated accumulation of high lipid content of 49.3% on peach. Hashem et al. [22] reported that watermelon peel waste was utilized for lipid biosynthesis by the oleaginous fungus *Lichtheimia corymbifera*, achieving a lipid content of 39.56%. Mhlongo et al. [9] achieved a lipid yield of 36% by cultivating *C. echinulata* ATHUM4411 on waste glycerol. By cultivation on orange peel, *C. echinulata* accumulated total lipid content of 46.6% that included 14.1% gamma-linolenic acid [52].

The differences observed in lipid biosynthesis among the tested agro-industrial substrates may be attributed to variations in their chemical composition and nutrient availability. Peach waste supported the highest lipid accumulation (49.30%), which may be due to its relatively high content of readily metabolizable sugars such as glucose, fructose, and sucrose that can be directly utilized by *C. echinulata* for biomass formation and fatty acid biosynthesis. In oleaginous fungi, excess carbon availability coupled with depletion of nitrogen promotes the redirection of carbon flux from cellular growth toward triacylglycerol synthesis, leading to enhanced lipid accumulation. Furthermore, peach residues may contain favorable levels of vitamins, minerals, and organic acids that support fungal metabolism and enzymatic activities involved in lipid biosynthesis [65]. By contrast, the comparatively lower lipid yields obtained from grape, apricot, plum wastes, and molasses may be associated with differences in their sugar composition, carbon-to-nitrogen ratio, or the presence of phenolic compounds and other metabolites that can partially inhibit fungal growth or lipid-producing enzymes [51].

GC-MS analysis of lipids extracted showed that the most abundant fatty acids identified were oleic acid, linoleic acid, gamma-linolenic acid, and palmitic acid, which accounted for over 83% of the total composition in all samples and ranged from 35.49% to 40.98%, 13.68% to 21.31%, 7.1% to 21.78% and 13.44% to 22.02%, respectively, across the analyzed samples. The observed differences in lipid composition among the samples could be attributed to the variations between natural and synthetic media. Natural media, such as the rotten peach fruit medium (A4), likely provide a complex array of nutrients and environmental conditions that influence the metabolic pathways of *C. echinulata*. This complexity led to a distinct fatty acid profile compared to synthetic media, typically designed to optimize specific growth parameters, and may lack the diversity of compounds found in natural sources. Consequently, the fatty acid profiles of the samples grown in synthetic media (A1, A2, and A3) were different from those grown in natural media, reflecting the impact of the growth environment on fatty acid synthesis and accumulation. Principal component analysis further supported this observation by showing high levels of similarity between the lipid extracts from *C. echinulata* cultivated in A1, A2, and A3 conditions, but A4 (peach wastes) showed minor differences in lipid composition. PCA was applied as a multivariate statistical approach to simplify the complex fatty acid dataset and to identify the major sources of variation among the analyzed lipid samples. The PCA results demonstrated clear discrimination among the lipid profiles based on differences in fatty acid composition. The first principal component (PC1) and second principal component (PC2) explained the majority of the total variance, indicating that these components effectively summarized the information contained in the original variables [66, 67]. Examination of the loading values revealed that the separation of samples was mainly influenced by variations in the relative abundances of the predominant fatty acids, particularly oleic acid, linoleic acid, γ-linolenic acid, and palmitic acid. Fatty acids located close to each other on the loading plot exhibited positive correlations, suggesting common biosynthetic regulation or similar responses to culture conditions. Conversely, fatty acids positioned in opposite directions were negatively correlated, indicating distinct metabolic allocation patterns [67]. The clustering pattern observed in the score plot suggests that cultivation conditions and substrate composition influenced the metabolic pathways involved in fatty acid biosynthesis, resulting in changes in lipid composition. Therefore, PCA provided a comprehensive visualization of the relationships among samples and fatty acid variables, confirming that the optimized cultivation conditions not only affected total lipid production but also influenced the qualitative fatty acid profile. This analysis highlights the value of PCA for evaluating lipid quality and identifying factors associated with desirable fatty acid compositions for potential pharmaceutical and nutraceutical applications [67, 68]. This analysis highlighted how agro-waste can significantly impact the lipid profile of fungi, leading to differences in lipid composition compared to synthetic cultures.

Papanikolaou et al. [69] reported that fermentation of *C. echinulata* on glucose can produce palmitic acid, stearic acid, oleic acid, linoleic acid, and *γ*-linolenic acid at a concentration of 17.2, 6.3, 49.4, 15.8, and 10.4%, respectively. Fakas et al. [70] demonstrated that *C. echinulata* exhibited an excellent fatty acid profile, including significant amounts of palmitic acid, stearic acid, oleic acid, linoleic acid, and γ-linolenic acid. Mohamed et al. [28] found that the fatty acid profiles of eleven Mucorales strains exhibited high fractions of SAFs, MUFAs, and PUFAs, including myristic, palmitic, palmitoleic, stearic, oleic, linoleic, and γ-linolenic acids. The diversity in fatty acids extracted from oleaginous fungi can be attributed to genetic variation among different fungal species and strains, significantly determining their unique metabolic pathways and the types of fatty acids they produce. Additionally, environmental conditions such as nutrient availability, temperature, pH, and oxygen levels during cultivation can significantly impact the fatty acid profiles of fungi. The type of substrate utilization also influences the synthesis and accumulation of specific fatty acids [71].

The results of the present study demonstrated that the crude lipids extracted from *C. echinulata* exhibited a broad spectrum of antimicrobial activity attributed to the high content of unsaturated fatty acids. These findings are consistent with those of Mohamed et al. [28], who reported that certain microbial lipid extracts from *Mucor* spp. showed antibacterial activity against *B. subtilis*, *S. aureus*, *E. coli*, and *S. typhimurium*, as well as antifungal activity against *(A) flavus* and *P. chrysogenum*. Firoozabad and Nasr [42] also indicated that extracted lipid from *C. echinulata* exhibited a high effect against *S. aureus*,* (B) cereus*,* B. subtilis*, and *E. coli*, with inhibition zones of 10.25, 8, 10.5, and 11 mm, respectively. The antimicrobial activity of fatty acids was stated to be dependent on chain length and unsaturation degree [72]. The antimicrobial activity observed in the present study can be correlated with the fatty acid composition of the lipid extract determined by GC–MS. The extract obtained under the best lipid-producing condition contained a high proportion of unsaturated fatty acids, particularly oleic acid, linoleic acid, and γ-linolenic acid, in addition to palmitic acid. Oleic acid represented the major fatty acid fraction, followed by γ-linolenic acid and linoleic acid, indicating that the extract was rich in long-chain unsaturated fatty acids. These fatty acids are amphipathic molecules capable of interacting with microbial cell envelopes and lipid bilayers. Recent studies have shown that long-chain unsaturated fatty acids such as oleic, linoleic, and linolenic acids can disturb bacterial membrane organization, induce membrane remodeling, increase permeability, and impair membrane-associated physiological processes [73].

The antimicrobial activity of fatty acids was due to their molecular structure and configuration. This includes factors such as the carbon chain length, double bond presence, number, position, and orientation. These structural characteristics determine how effectively the fatty acid can interact with and disrupt microbial cell membranes [74]. Mandey et al. [75] found that medium and long-chain unsaturated fatty acids appear more effective against Gram-positive than Gram-negative bacteria. This difference may be due to variations in cell wall composition, which allow better access to the bacterial membrane in Gram-positive bacteria than in Gram-negative bacteria [76].

## Conclusions

Soil-derived *C. echinulata* HAA strain introduced oleaginous potential of 61.23% under OFAT conditions (30 °C, spore concentration of 10⁴ spores/mL, glucose (60 g/L), ammonium tartrate (2 g/L), and C/N ratio of 78.4, at pH 7 for 7 days) compared to 55.37% lipid content for the original fermentation basal medium. Statistical optimization by RSM-BBD presented lipid content of 59.37%. In addition, cultivation of *C. echinulata* on agro-wastes produced 49.30% lipid content with peach. Comparative GC-MS analysis of the best lipid yield conditions revealed different fatty acids, with high percentages oleic acid, linoleic acid, gamma-linolenic acid, and palmitic acid. The extracted lipids showed reasonable antimicrobial activity against pathogenic bacteria and *Candida* strains. This study concludes that *C. echinulata* attaining interest as convenient lipid producer to be utilized sustainably.

## Supplementary Information

Below is the link to the electronic supplementary material.

Supplementary Material 1

## Data Availability

The dataset generated and/or analyzed during the current study is available in the GenBank.
