# Supplementary material for: Amelioration of lipid production by Cunninghamella echinulata: statistical optimization and GC-MS characterization
Source: Microb Cell Fact. 2026 Nov 3;25:230. doi: 10.1186/s12934-026-03134-3 (PMC13737615; doi:10.1186/s12934-026-03134-3)
Supplement: Supplementary file 1 [file 12934_2026_3134_MOESM1_ESM.docx]

**Supplementary**

**Fig. 1S** Standard curve of sugar from D-glucose.

**Fig. 2S** Standard curve of canola oil.

**Fig. 3S** Percent of similarity and divergence of the phylogenetic tree of *C. echinulata* HAA. Percent similarity in the upper triangle and percent divergence in the lower triangle.

**Fig. 4S** Pareto chart and normal probability plot showing the effects of the investigated factors on lipid content of *C. echinulata*.

**Fig. 5S** Chemical structure of fatty acids detected in lipid extracts of *C. echinulata*.

**Table 1S** GC-MS chemometric profile of the saponifiable lipid extracts from *C. echinulata*.

| **Peak** | **Name** | **Retention time** | **Area %** | | | |
| --- | --- | --- | --- | --- | --- | --- |
|  |  |  | **A1** | **A2** | **A3** | **A4** |
| 1 | Myristic acid  **C_14_H_28_O_2_ (C14:0)** | 21.277 | 0.41 | 0.49 | 0.48 | 0.67 |
| 2 | Pentadecanoic acid  **C_15_H_30_O_2_ (C15:0)** | 24.147 | 0.17 | 0.14 | 0.13 | 0.04 |
| 3 | Palmitic acid  **C_16_H_32_O_2_ (C16:0)** | 26.954 | 13.44 | 14.41 | 14.14 | 22.02 |
| 4 | Palmitoleic acid  **C_16_H_30_O_2_ (C16:1)** | 28.132 | 1.2 | 1.07 | 1.12 | 0.64 |
| 5 | Margaric acid  **C_17_H_34_O_2_ (C17:0)** | 29.729 | 0.09 | 0.09 | 0.07 | 0.05 |
| 6 | *Cis*-10-Heptadecenoic acid **C_17_H_32_O_2_ (C17:1)** | 30.703 | 0.14 | 0.11 | 0.12 | 0.04 |
| 7 | Stearic acid  **C_18_H_36_O_2_ (C18:0)** | 32.39 | 2.02 | 3.65 | 3.12 | 10.11 |
| 8 | Oleic acid  **C_18_H_34_O_2_ (C18:1)** | 33.272 | 35.49 | 38.07 | 39.18 | 40.98 |
| 9 | Linoleic acid  **C_18_H_32_O_2_ (C18:2)** | 34.975 | 21.31 | 18.84 | 18.37 | 13.68 |
| 10 | Gamma-Linolenic acid **C_18_H_30_O_2_ (C18:3)** | 36.195 | 21.78 | 18.69 | 19.3 | 7.1 |
| 11 | Arachidic acid  **C_20_H_40_O_2_ (C20:0)** | 37.464 | 0.12 | 0.2 | 0.18 | 0.62 |
| 12 | *Cis*-11-Eicosenoic acid **C_20_H_38_O_2_ (C20:1)** | 38.171 | 0.88 | 0.88 | 0.85 | 0.4 |
| 13 | *Cis*-11,14-Eicosadienoic acid  **C_20_H_36_O_2_ (C20:2)** | 39.767 | 0.36 | 0.33 | 0.3 | 0.11 |
| 14 | Homo-γ-linolenic acid **C_20_H_34_O_2_ (C20:3)** | 40.843 | 0.07 | 0.06 | 0.06 | 0.02 |
| 15 | Behenic acid  **C_22_H_44_O_2_ (C22:0)** | 42.255 | 0.27 | 0.39 | 0.34 | 0.86 |
| 16 | Erucic acid  **C_22_H_42_O_2_ (C22:1)** | 42.865 | 0.11 | 0.11 | 0.1 | 0.04 |
| 17 | Lignoceric acid  **C_24_H_48_O_2_ (C24:0)** | 46.794 | 1.57 | 1.89 | 1.65 | 2.37 |
| 18 | Nervonic acid  **C_24_H_46_O_2_ (C24:1)** | 47.305 | 0.17 | 0.24 | 0.18 | 0.08 |
| 19 | Docosahexaenoic acid **C_22_H_32_O_2_ (C22:6)** | 48.95 | 0.39 | 0.35 | 0.3 | 0.16 |

**A1**: basal medium, **A2**: OFAT optimized medium, A3: statistically optimized medium, and **A4**: peach waste medium.

**Table 2S** Antimicrobial activity of lipid extracts from *C. echinulata* against pathogenic microorganisms.

| **Extracted lipids** | Inhibition zone diameter (mm) | | | | | | | |
| --- | --- | --- | --- | --- | --- | --- | --- | --- |
|  | ***B. subtilis*** | ***S. aureus*** | ***E. coli*** | ***S. typhi*** | ***C. albicans*** | ***C. glabrata*** | ***A. flavus*** | ***A. niger*** |
| **A1** | 13±0.92^c^ | 17±0.82^c^ | 16±0.81^d^ | 0.0±0.0^b^ | 13±0.81^b^ | 12±0.62^c^ | 0.0±0.0^b^ | 0.0±0.0^b^ |
| **A2** | 9±0.53^e^ | 14±0.28^d^ | 16±0.57^d^ | 0.0±0.0^b^ | 13±0.54^b^ | 11±0.43^d^ | 0.0±0.0^b^ | 0.0±0.0^b^ |
| **A3** | 15±0.75^b^ | 17±0.23^c^ | 19±0.36^b^ | 0.0±0.0^b^ | 13±0.60^b^ | 14±0.43^b^ | 0.0±0.0^b^ | 0.0±0.0^b^ |
| **A4** | 11±0.47^d^ | 18±0.21^b^ | 17±0.40^c^ | 0.0±0.0^b^ | 13±0.26^b^ | 11±0.31^d^ | 0.0±0.0^b^ | 0.0±0.0^b^ |
| **Chloramphenicol** | 27±0.94^a^ | 28±0.81^a^ | 28±0.47^a^ | 21±0.41^a^ | 0.0±0.0^c^ | 0.0±0.0^e^ | 0.0±0.0^b^ | 0.0±0.0^b^ |
| **Fluconazole** | 0.0±0.0^f^ | 0.0±0.0^e^ | 0.0±0.0^e^ | 0.0±0.0^b^ | 20±0.94^a^ | 19±0.47^a^ | 17±0.47^a^ | 17±0.47^a^ |

**Table 3S** Minimum inhibitory concentration of lipid extracts against tested microorganisms.

| **Lipid extracts** | **MIC (mg/mL)** | | | | |
| --- | --- | --- | --- | --- | --- |
|  | ***B. subtilis*** | ***S. aureus*** | ***E. coli*** | ***C. albicans*** | ***C. glabrata*** |
| **A1** | 25 | 25 | 25 | 50 | 25 |
| **A2** | 50 | 25 | 12.5 | 25 | 25 |
| **A3** | 25 | 12.5 | 12.5 | 50 | 25 |
| **A4** | 25 | 25 | 12.5 | 50 | 50 |
| **Chloramphenicol** | 3.125 | 6.25 | 6.25 | ND | ND |
| **Fluconazole** | ND | ND | ND | 12.5 | 6.25 |

**ND:** not detected
